# Description of electroneuromiographic and laboratorial findings in leprosy neuropathy, according to its clinical forms: the confirmation of a spectral disease

**DOI:** 10.3389/fmed.2023.1304131

**Published:** 2024-01-08

**Authors:** Diogo Fernandes dos Santos, Isabella Sabião Borges, Leonardo Peixoto Garcia, Douglas Eulálio Antunes, Andrea De Martino Luppi, Isabela Maria Bernardes Goulart

**Affiliations:** ^1^National Reference Center for Sanitary Dermatology and Leprosy, Clinics’ Hospital, School of Medicine, Federal University of Uberlândia (UFU), Uberlândia, Brazil; ^2^Postgraduate Program in Health Sciences, School of Medicine, Federal University of Uberlândia (UFU), Uberlândia, Brazil; ^3^Radiology Division, Clinics’ Hospital, School of Medicine, Federal University of Uberlândia (UFU), Uberlândia, Brazil

**Keywords:** leprosy, peripheral nerves/neurophaty, electroneuromyography, neuropathy, *Mycobacterium leprae*, Hansens’s disease

## Abstract

**Introduction:**

Leprosy is one of the most common infectious cause of peripheral neuropathy in the world and can lead to sequelae and physical disabilities. Electroneuromyography (ENMG) is the gold-standard test for evaluating neural impairment, detecting from subclinical abnormalities to advanced lesions. This study aims to describe the electroneuromyographic findings in patients with leprosy, according to their clinical forms.

**Methods:**

The study is a retrospective observational analysis of the medical records of patients with leprosy, of a National Reference Center of Sanitary Dermatology and Leprosy in Brazil between 2014 and 2022. 513 patients underwent ENMG at leprosy diagnosis and also underwent a clinical, serological and molecular evaluation of the disease.

**Results:**

The electroneuromyographic findings showed 2,671 altered nerves, with an average of 6.9 (±5.1) altered nerves per patient. The most affected sensory nerves were the superficial peroneal (25.0%; 413/1649), sural (15.1%; 397/2627) and ulnar (13.8%; 363/2627), with average of 4.3 (±3.2) affected sensory nerves per patient. The most affected motor nerves were the ulnar (33.1%; 338/1022) and common peroneal (12.1%; 319/2627), with average of 2.6 (±2.5) motor nerves affected per patient. 126 patients presented normal ENMG and, among the 387 with abnormalities in the exam, 13.2% (51/387) had mononeuropathy and 86.8% (336/387) had multiple mononeuropathy. Axonal involvement was more frequent in primary neural leprosy, borderline-tuberculoid, borderline-lepromatous and lepromatous forms.

**Discussion:**

Our findings support that leprosy is a spectral disease, characterized by a balance between host immunity and bacillary load. Therefore, the impairment and electroneuromyographic characteristics are distinct and may vary according to the clinical form.

## Introduction

Leprosy is one of the oldest diseases of humanity and still one of the most neglected today (1, 2). The disease, which affects nerves, skin and other tissues, is one of the most common infectious cause of peripheral neuropathy worldwide and can lead to sequelae and physical disabilities that perpetuate prejudice and stigma linked to the condition (3).

The gold standard for diagnosing leprosy is performed by dermato-neurological clinical examination and bacilloscopy of skin lesion biopsy and slit skin smears, however, this method does not detect the paucibacillary and primary neural forms, because they have a small number of bacilli (4). Therefore, the use of serological and molecular tools has become essential in the early diagnosis of leprosy, as well as in other chronic infectious diseases (5, 6). Furthermore, peripheral neural impairment is not always recognized in the diagnosis, although it can occur in any form of the disease and is the main cause of sequelae and disabilities (7, 8).

Leprosy is classified into different clinical forms according to the Ridley-Jopling classification, proposed in the 1960s, which is based on the host’s immune response, histopathological classification of the skin lesion and bacillary load. According to this classification, patients with a better cellular-type immune response against *Mycobacterium leprae* (mediated by T-lymphocytes) are classified as tuberculoid (T), while anergic patients with an important deficiency of this type of cellular immune response are classified as lepromatous (L) (9–11).

Between these two extremes, there are patients defined as borderline, that present intermediate immunological responses. In according to the unstable immune response of this intermediate form, they can be differentiated as borderline-lepromatous (BL), borderline-tuberculoid (BT) and borderline-borderline (BB) (9–11).

Clinically, the T form is shown as few or single lesions with well-defined borders, with different sizes, presenting anesthesia or hypoesthesia within the lesion, often accompanied by a neural trunk impairment, even with neural abscesses in more severe cases. The BT form is similar to the T form, but presents annular macular with raised poorly defined borders, also with asymmetric and intense neural impairment. BB is clinically identified by the presence of lesions with irregular contours, a ferruginous hue with a flat, smooth, circular center with an infiltrated periphery and a thick foveolar border, also with intense and extensive neural lesion (9–11).

The BL group is analogous to the L group, since they present numerous brownish lesions, with few neural lesions, but with the presence of visceral involvement. Group L manifests itself with diffuse and also visceral lesions, with cutaneous infiltration forming papules, hansenomas, plaques with a ferruginous aspect. In late cases, it may be observed several signs and symptoms such as leonine facies, madarosis, perforation of the nasal septum, osteoarticular lesions (lytic lesions), periodontal lesions, laryngitis (hoarseness and dyspnea), hepatic, splenic, and testicular involvement, among others (9–11).

In some patients, leprosy may manifest as a clinical presentation of the disease without any skin lesions, defined as primary neural leprosy (PNL). By definition, PNL refers to cases in which the presence of a peripheral neuropathy is observed, which may present as a mononeuropathy, multiple mononeuropathy or even a confluent multiple mononeuropathy, in the absence of skin lesions on dermaneurological examination and in which bacilloscopy of the slit skin smear is negative (5, 6).

Peripheral nerve involvement can occur in all clinical forms of leprosy, but it has not yet been described in detail according to serological, molecular and neurophysiological studies. Leprosy is a spectral disease and, consequently, neural damage is diverse and can manifest with different phenotypes, under the strong influence of the time from diagnosis to disease presentation (6). Thus, this study’s main objective is to describe the electroneuromyographic and laboratorial findings in patients with leprosy, according to their clinical forms.

## Methods

### Ethics statement

The research was submitted and approved by the Ethics Committee of the Federal University of Uberlandia (CAEE 45007721.7.0000.5152).

### Patients

The study was carried out through a retrospective observational evaluation of the medical records of patients with leprosy regularly followed up at the National Reference Center of Sanitary Dermatology and Leprosy (CREDESH), at the Clinical Hospital (HC), at the Faculty of Medicine of the Federal University of Uberlândia, who performed electroneuromyography (ENMG) in the period between 2014 and 2022.

The study adopted, as inclusion criteria, patients with the clinical diagnosis of leprosy, who were submitted to the ENMG examination during the diagnosis. Patients with other possible etiologies for a peripheral neuropathy were excluded, including: chronic alcoholism, diabetes mellitus, thyroid disease, malnutrition, hereditary neuropathy, hepatitis B or C, human immunodeficiency virus (HIV) and rheumatological diseases (e.g., rheumatoid arthritis, systemic lupus erythematosus, Sjogren’s and sarcoidosis).

### Clinical characterization

Epidemiological data (age, gender, previous contact with leprosy patients) and clinical data (symptoms, clinical forms of the disease, sensory impairment, presence of muscle weakness and amyotrophy, presence of neural thickening) were evaluated according to the data obtained from the medical records. The Ministry of Health protocol was used to assess the patient’s level of functional disability during diagnosis and at the end of treatment, which assesses the degree of physical disability and the integrity of neural function through muscle strength and sensitivity tests (12).

### Laboratory analyses

#### Bacilloscopy

The bacillary load analysis was performed on slit skin smears from six sites (both ear lobes, elbows and knees) and also on samples of skin and/or nerve biopsies, according to clinical evaluation. The result was described according to the number of bacilli found in an average microscopic field.

#### Anti-PGL-I ELISA serological test

Serum IgM antibodies were detected using the enzyme-linked immunosorbent assay (ELISA) performed against the native molecule PGL-I purified from the *Mycobacterium leprae* cell wall, the disease agent, according to a methodology previously described (5, 13). Antibody titer values above 1.0 were considered positive.

#### DNA extraction and real-time quantitative PCR

DNA was extracted from dermal smear samples and biopsies (nerve and overlying skin) and detected by quantitative real-time polymerase chain reaction (qPCR) primer/probe assay targeting the *M. leprae* species-specific genomic region (RLEP3) (14). The reactions were performed on the ABI7300 platform (Applied Biosystems) and the result was analyzed using the 7,300 System SDS Software vs. 1.4.

### Electroneuromyography

The electroneuromyographic study was performed using the MEB 4200 K electroneuromyograph (NIHON-KODHEN). In the study of sensory conduction, the median, ulnar, radial, sural and superficial fibular nerves were examined bilaterally. The motor conduction study evaluated the median, ulnar, common fibular and tibial nerves bilaterally, complemented by the identification of sites often involved in leprosy neuropathy, such as median nerve at the wrist, ulnar nerve at the elbow, fibular nerve at the fibular head and tibial nerve at the ankle. In some cases, according to the clinical evaluation, other nerves were included in the analysis. The parameters used to evaluate each nerve are described separately as a supplementary file.

Together with the affected nerves, the following data obtained from the medical records were collected and evaluated: number of sensory nerves affected per patient, number of motor nerves affected per patient and neurophysiological pattern (mononeuropathy x multiple mononeuropathy).

The presence of sensory axonal impairment was defined by the presence of a reduced sensory nerve action potentials (SNAP) amplitude (below reference values or greater than 50% SNAP amplitude asymmetry comparing to the contralateral side), while the motor axonal impairment was defined by an amplitude’s reduction of the compound muscle action potentials (CMAP).

Demyelinating impairment was considered when one of the following criteria was present: Prolonged distal motor latency ≥50% above the upper limit of normal; reduction in motor conduction velocity ≥30% below the lower limit of normal; conduction block defined by a ≥50% reduction in the negative proximal amplitude of peak evoked amplitude proximal relative to distal motor function and presence of abnormal temporal dispersion (>30% increase in the duration between the distal and proximal negative peak of the motor evoked potential).

The neurophysiological pattern was defined as: (i) focal demyelinating mononeuropathy; (ii) axonal sensory mononeuropathy; (iii) asymmetrical demyelinating neuropathy; (iv) asymmetrical axonal neuropathy; or (v) asymmetrical axonal neuropathy with focal slowing of conduction velocity.

### Skin biopsy

The skin biopsy site was determined according to the clinical assessment of each patient. Due to the absence of skin lesions in cases of primary neural leprosy, the skin biopsies of these patients were performed in the region close to the elbow, as it is a cold area and with possible intradermal involvement, since *M. leprae* has tropism for places with lower temperatures (5, 6, 15). Fite-Faraco stain was performed for bacilli identification.

### Peripheral nerve biopsy

The selection of peripheral nerves for biopsy was guided according to the patient’s clinical condition, with the selection of nerves that presented unilaterally or bilaterally decreased amplitude of SNAP, considering reference values; relative reduction in measurements of SAP amplitudes (over 50% decrease compared with the contralateral side); change in sensitivity and/or thickening; and electrophysiological abnormalities such as unilaterally or bilaterally unresponsiveness.

The selected branches were purely sensory, in order to minimize any damage to the patient, who was warned about this and about the fact that, due to the procedure, there would be no damage to any motor function. Together, local anesthesia was applied to the area to be biopsied and, during the procedure, the nerve was fasciculated and completely sectioned. The samples were processed and studied according to routine standard procedures (5, 15).

### Statistical analysis

Continuous and dichotomous variables were applied to evaluate differences of clinical, electromyographic and laboratory factors between groups. The Kolmogorov–Smirnov test was applied to verify data normality prior analyses. The Wilcoxon-Mann–Whitney U-test was carried out to compare differences between independent groups when the dependent variables were not normally distributed. The binomial test was applied to evaluate dichotomous variables. The chi-square test was used to analyze associations between clinical, laboratorial and electromyographic abnormalities and the clinical forms of leprosy. The statistical software used was GraphPad Prism version 7 (La Jolla, CA, United States), and all tests presenting a probability below 5% were considered significant.

## Results

Five hundred and thirteen medical records of leprosy patients regularly monitored at a national leprosy reference center in Brazil, who underwent electroneuromyography (ENMG) in the period between 2014 and 2022, were analyzed. The patients’ median age was 45.8 years (± 16.6), with 50.1% male (257/513; Table 1).

**Table 1:** Epidemiological, clinical and laboratory characteristics among the leprosy patients.

	Leprosy patients
	*n* = 513
Age	45,8 ± 16,6
Sex	
Male	50,1% (257/513)
Female	49,9% (256/513)
Clinical form	
Primary Neural Leprosy	20,9% (107/513)
Tuberculoid	1,0% (5/513)
Borderline-tuberculoid	53,6% (275/513)
Borderline-Borderline	6,2% (32/513)
Borderline-lepromatous	7,2% (37/513)
Lepromatous	11,1% (57/513)
Reactional states	
Type 1	34,9% (179/513)
Type 2	15,8% (81/513)
WHO leprosy-related impairment	
0	52,8% (271/513)
1	24,4% (125/513)
2	22,8% (117/513)
ELISA anti-PGLI	57,9% (297/513)
ELISA index	2,31 ± 1,03
Slit skin smear	
Bacilloscopy	21,0% (108/513)
qPCR	51,3% (263/513)
Skin biopsy	
Bacilloscopy	18,5% (95/513)
qPCR	47,2% (242/513)
Histopathology	20,3% (104/513)
Nerve Biopsy	
Bacilloscopy	6,7% (5/75)
qPCR	37,3% (28/75)
Histopathology	9,3% (7/75)

n, number of leprosy patients; WHO, World Health Organization; ELISA, enzyme-linked immunosorbent assay; anti-PGL-1, anti-phenolic glycolipid-1; qPCR, Real Time Quantitative Polymerase Chain Reaction.

### Clinical characterization

Regarding the clinical forms of leprosy, there was a predominance of the BT form (53.6%; 275/513), followed by PNL (20.9%; 107/513) and L (11.1%; 57/513). 7.2% (37/513) of the patients had the borderline-lepromatous form, 6.2% (32/513) the BB form, and only 1% (5/513) the T form. Regarding the operational classification for treatment purposes, multibacillary (MB) patients accounted for 85.8% (440/513) of cases, while paucibacillary (PB) patients accounted for 14.2% (73/513). At clinical presentation, sensory symptoms were present in 73.3% (376/513) of the cases in total, and motor symptoms in 49.3% (253/513) of them (Table 1).

Neural thickening of one or more nerves was observed in 71.7% (368/513) of patients, with an average of 3.5 (±1.8) thickened nerves per patient. As for the disability evaluation at diagnosis according to the Word Health Organization (WHO) disability score, 52.8% (271/513) had grade 0 disability, while 24.4% (125/513) of patients had grade 1 and 22.8% (117/513) grade 2 disability (Table 1).

Regarding the occurrence of reactional states, 50.7% (260/513) of the patients had at least one type of leprosy reaction, with type 1 being the most frequent of them (34.9%; 179/513), followed by 15.8% (81/513) with type 2 reaction (Table 1).

### Laboratory analysis

The anti-PGLI IgM ELISA serology was positive in 57.9% (297/513) of the patients, with an index of 1.6 (±1.7). The qPCR test of slit skin smear was positive in 51.7% (265/513) of the cases, a result superior to that of the bacilloscopy, whose positivity was only 21.0% (108/513). Regarding the skin biopsy performed in all cases, the qPCR test was also superior to bacilloscopy, being positive in 47.2% (242/513) and 18.5% (95/513) of cases, respectively. The superiority of the qPCR result was also seen in peripheral nerve samples, with positivity of 37.3% (28/75) of patients, and only 6.7% (5/75) of positivity in bacilloscopy. Skin biopsy showed histopathological abnormalities in 20.3% (104/513) of the cases in total, while only 9.3% (7/75) of the nerves that underwent biopsy showed a definitive histopathological abnormality of leprosy (Table 1).

### Electroneuromyographic findings

Regarding the electroneuromyographic evaluation, 28.1% (144/513) did not present any type of impairment related to leprosy and 13.3% (68/513) presented other abnormalities such as carpal tunnel syndrome and radiculopathy. In these cases, the diagnosis was based on the dermatological abnormalities observed in the disease. 71.9% (369/513) had a neural impairment compatible with leprosy neuropathy on the ENMG.

In total, 2,627 affected nerves were found, with an average of 7.1 (±5.2) nerves per patient. The most affected sensory nerves were the superficial peroneal nerve (15.8%; 413/2627), followed by the sural (15.1%; 397/2627), ulnar (13.8%; 363/2627); median (9.9%; 261/2627) and radial (6.9%; 181/2627), with a mean of 4.4 (±3.3) sensory nerves affected per patient. The most affected motor nerves were the ulnar (12.9%; 338/2627), followed by the common peroneal (12.1%; 319/2627), tibial (6.8%; 180/2627) and median (6.7%; 174/2627), with an average of 2.7 (±2.5) affected motor nerves per patient. Table 2 shows the most affected sensory and motor nerves in the electroneuromyographic evaluation.

**Table 2:** Distribution of the most affected peripheral nerves in leprosy patients.

Affected nerve	n	%
Sensorial	1,615	61,5%
Median	261	9,8%
Ulnar	363	13,6%
Radial	181	6,8%
Sural	397	14,9%
Superficial peroneal	413	15,5%
Motor	1,012	38,5%
Median	175	6,6%
Ulnar	338	12,6%
Tibial	180	6,7%
Peroneal	319	11,9%
Total nerves	2,627	100%

n, number of nerves; %, percentages of nerves.

Among patients with neural impairment compatible with leprosy neuropathy, 19.2% (71/369) had only one altered nerve (mononeuropathy), while 80.8% (298/369) had two or more altered nerves (asymmetrical multiple mononeuropathy). The electroneuromyographic pattern and its distribution are detailed in Table 3.

**Table 3:** Distribution of the electroneuromyographic pattern in patients with leprosy.

Electroneuromyographic pattern	*n*	%
Focal demyelinating mononeuropathy	35	9,5
Sensory axonal mononeuropathy	36	9,8
Asymmetrical demyelinating neuropathy	30	8,1
Asymmetrical axonal neuropathy	69	18,7
Asymmetrical neuropathy with focal slowing of conduction velocity	199	53,9
Total	369	100

n, number of electroneuromyographic pattern; %, percentages of electroneuromyographic pattern.

Table 4 presents the distribution of patients with leprosy according to their electroneuromyographic pattern, comparing the laboratory and clinical characteristics of patients with pattern of mononeuropathy and multiple mononeuropathy.

**Table 4:** Distribution of patients with leprosy according to the electroneuromyographic pattern.

Parameters	Mononeuropathy	Multiple mononeuropathy	*p* value
Neural thickening	46,5% (33/71)	89,3% (266/298)	< 0.0001
Sensory symptoms	52,1% (37/71)	90,6% (270/298)	< 0.0001
Motor symptoms	40,8% (29/71)	61,1% (182/298)	0.0020
WHO leprosy-related impairment			
0	70,4% (50/71)	33,5% (100/298)	< 0.0001
1	12,7% (9/71)	31,9% (95/298)	0.0012
2	16,9% (12/71)	34,6% (103/298)	0.0039
Type I reactional state	28,2% (20/71)	45,6% (136/298)	0.0074
Type II reactional state	1,4% (1/71)	25,2% (75/298)	< 0.0001
ELISA anti-PGLI	54,9% (39/71)	58% (173/298)	0.6323
ELISA anti-PGLI index	1,2 (± 0,9)	1,9 (± 2,0)	0.1545
Slit skin smear qPCR	50,7% (36/71)	50,3% (150/298)	0.8749
Skin biopsy qPCR	40,8% (29/71)	51% (152/298)	0.1238
Nerve biopsy qPCR	42,8% (9/21)	79,2% (19/54)	0.5374
Slit skin smear bacilloscopy	5,6% (4/71)	30,2% (90/298)	< 0.0001
Skin biopsy bacilloscopy	5,6% (4/71)	27,2% (81/298)	0.0001
Nerve biopsy bacilloscopy	4,8% (1/21)	16,7% (4/54)	0.6801
Skin biopsy histopathology	12,7% (9/71)	25,8% (77/298)	0.0184
Nerve biopsy histopathology	9,5% (2/21)	20,8% (5/54)	0.9718

WHO, World Health Organization; ELISA, enzyme-linked immunosorbent assay; anti-PGL-1, anti-phenolic glycolipid-1; qPCR, Real Time Quantitative Polymerase Chain Reaction.

Patients with multiple mononeuropathy had a higher prevalence of sensory-motor impairment and neural thickening. This group also showed a higher prevalence of leprosy reaction and a greater severity of neural impairment documented by the degree of disability. Regarding the laboratory findings, there was a greater positivity in the evaluation by bacilloscopy of the slit skin smear and skin biopsy, which also showed more histopathological abnormalities. Molecular evaluations showed no difference between the groups, although they showed significant positivity in all evaluations when compared with bacilloscopy (Table 4).

### Clinical, laboratory and electroneuromyographic evaluation by leprosy clinical forms

Clinical, laboratory and electroneuromyographic data of patients with leprosy were also evaluated according to the different clinical forms and are presented in Table 5, confirming the spectral characteristic of leprosy neuropathy.

**Table 5:** Clinical, laboratory and electrophysiological findings of patients with leprosy.

Parameters	PNL	T	BT	BB	BL	L	*χ^2^*
Clinical data							Value of *p*
Neural thickening	68.2% (73/107)	20% (1/5)	64.7% (178/275)	87.5% (28/32)	100% (37/37)	89.5% (51/57)	0.0005
Sensory symptoms	82.2% (88/107)	20% (1/5)	63.3% (174/275)	81.2% (26/32)	89.2% (33/37)	94.7% (54/57)	0.0071
Motor symptoms	64.5% (69/107)	40% (2/5)	40.4% (111/275)	43.7% (14/32)	54.0% (20/37)	64.9% (37/57)	0.0831
Number of skin lesions							
0:	100% (107/107)	0% (0/5)	47.3% (130/275)	18.7% (6/32)	18.9% (7/37)	14.0% (8/57)	< 0.0001
1–4:	–	100% (5/5)	36% (99/275)	25.0% (8/32)	10.8% (4/37)	10.5% (6/57)	< 0.0001
≥ 5	–	0% (0/5)	16.7% (46/275)	56.3% (18/32)	70.3% (26/37)	75.5% (43/57)	0.0046
WHO leprosy-related impairment							
0	42.1% (45/107)	80% (4/5)	65.4% (180/275)	43.7% (14/32)	37.8% (14/37)	24.6% (14/57)	0.0009
1	16.8% (18/107)	20% (1/5)	18.9% (52/275)	31.3% (10/32)	35.1% (13/37)	54.4% (31/57)	0.0024
2	41.1% (44/107)	0% (0/5)	15.6% (43/275)	25.0% (8/32)	27.1% (10/37)	21.0% (12/57)	< 0.0001
Type I reactional state	34.6% (37/107)	40% (2/5)	29.8% (82/275)	53.1% (17/32)	64.9% (24/37)	29.8% (17/57)	< 0.0001
Type II reactional state	0.9% (1/107)	0% (0/5)	2.9% (8/275)	9.4% (3/32)	51.3% (19/37)	87.7% (50/57)	< 0.0001
Laboratory data							
ELISA anti-PGLI	32.7% (35/107)	20% (1/5)	59.3% (163/275)	65.6% (21/32)	78.4% (29/37)	84.2% (48/57)	< 0.0001
ELISA anti-PGLI index	0.9 (± 0.9)	0.6 (± 0.4)	1.4 (± 1.3)	1.9 (± 1.7)	2.4 (± 1.7)	3.5 (± 2.7)	< 0.0001
Slit skin smear qPCR	37.4% (40/107)	–	52.4% (144/275)	40.6% (13/32)	62.2% (23/37)	78.9% (45/57)	< 0.0001
Skin biopsy qPCR	26.2% (28/107)	–	41.0% (113/275)	59.4% (19/32)	86.5% (32/37)	87.7% (50/57)	< 0.0001
Nerve biopsy qPCR	32.2% (20/62)	–	61.5% (8/13)	0% (0/0)	0% (0/0)	0% (0/0)	0.0629
Slit skin smear bacilloscopy	–	–	7.6% (21/275)	53.1% (17/32)	67.6% (25/37)	75.4% (43/57)	< 0.0001
Skin biopsy bacilloscopy	4.7% (5/107)	–	8.0% (22/275)	37.5% (12/32)	59.5% (22/37)	59.6% (34/57)	< 0.0001
Nerve biopsy bacilloscopy	6.4% (4/62)	–	7.7% (1/13)	–	–	–	< 0.0001
Skin biopsy histopathology	3.7% (4/107)	20% (1/5)	13.8% (38/275)	40.6% (13/32)	62.2% (23/37)	43.8% (25/57)	< 0.0001
Nerve biopsy histopathology	8.1% (5/62)	–	15.4% (2/13)	–	–	–	< 0.0001
Electroneuromyographic data							
Abnormal ENMG	94.4% (101/107)	60% (3/5)	55.3% (152/275)	75% (24/32)	91.9% (34/37)	96.5% (55/57)	< 0.0001
Number of altered nerves	6.9 (± 5.2)	1.0(± 0.0)	5.9 (± 5.2)	8.5 (± 5.6)	10.1 (± 4.1)	7.7 (± 4.3)	< 0.0001
Mononeuropathy	24.7% (25/101)	100% (3/3)	25% (38/152)	8.3% (2/24)	2.9% (1/34)	3.6% (2/55)	< 0.0001
Multiple mononeuropathy	75.3% (76/101)	–	75% (114/152)	91.7% (22/24)	97.1% (33/34)	96.4% (53/55)	
Sensory axonal impairment	88.1% (89/101)	66.7% (2/3)	81.6% (124/152)	100% (24/24)	100% (34/34)	100% (55/55)	0.8201
Motor axonal impairment	47.5% (48/101)	33.3% (1/3)	37.5% (57/152)	50% (12/24)	55.9% (19/34)	40.0% (22/55)	0.0722
Demyelinating impairment	85.1% (86/101)	100% (3/3)	78.9% (120/152)	75% (18/24)	79.4% (27/34)	70.9% (39/55)	0.0084

n, number of leprosy patients; WHO, World Health Organization; ELISA, enzyme-linked immunosorbent assay; anti-PGL-1, anti-phenolic glycolipid-1; qPCR, Real Time Quantitative Polymerase Chain Reaction; ENMG, electroneuromyography; PNL, primary neural leprosy; T, tuberculoid; BT, borderline-tuberculoid; BB, borderline-borderline; BL, borderline-lepromatous; L, lepromatous.

The presence of neural thickening was very prevalent in all clinical forms, with the exception of the T group. In all clinical forms, there was a predominance of sensory symptoms, especially in the PNL, BB, BL and L forms. There was a greater balance in relation to motor impairment (Table 5).

Patients in the PNL group had the highest proportion of individuals with grade 2 disability at the time of diagnosis [41.1%; (44/107)]. The presence of type I leprosy reaction was more prevalent in the borderline groups (BB and BL), while the presence of leprosy erythema nodosum was more common in group L (Table 5).

The serological evaluation by the anti-PGLI ELISA confirmed a higher positivity in the borderline group (BT, BB and BL) and L, including with an ascending pattern of the ELISA index. Molecular evaluation using slit skin smear and skin biopsy qPCR also showed a higher bacillary load from the borderline group (BT, BB and BL) and L. The histopathological evaluation of the skin showed greater positivity in individuals from the BB, BL and L groups (Table 5).

Electroneuromyographic abnormalities were more frequent in the PNL, BB, BL and L groups. There was a predominance of abnormalities in the sensory conduction study in relation to motor conduction in all groups. The predominance of sensory impairment was confirmed both by the proportion of patients with sensory impairment and the number of nerves affected per patient. In addition, it is noteworthy that, with the exception of the T group, the most prevalent impairment is sensory and axonal, followed by focal demyelination and, finally, motor axonal impairment. All groups had a higher proportion of multiple mononeuropathy, with the exception of group T, which had the mononeuropathy pattern in all evaluated patients. The electroneuromyographic pattern of an asymmetrical axonal neuropathy with focal slowing of conduction velocity was the most common in PNL, BT, BB, BL and L (Table 5).

## Discussion

*Mycobacterium leprae* is an acid-fast bacillus with very slow growth and multiplication. Its pathogenetic action is due to the impairment of the truncal and intradermal peripheral nerves, for which it has a marked affinity, and to the host’s response, which is extremely variable. The cellular mediated immunity plays a key role in leprosy as it protects the individual against the disease, since this response to *M.leprae* antigenens determines the host’s position in the disease spectrum. Traditionally, it is still recognized as a skin disease, whose lesions and hypochromic macules, accompanied by sensory deficits, have become the key to diagnosis. However, it is known that regardless of the clinical form, leprosy is primarily a neural disease and the recognition of peripheral nerve impairment has become mandatory in all clinical forms (16–18).

Clinically, neural damage in leprosy is asymmetrical and characterized by the presence of peripheral nerve hypertrophy and predominantly sensory symptoms. However, there is an evident polymorphism in this disease, including sensory impairment, which makes timely recognition and treatment difficult. Motor impairment, in addition to being later, is more observed in borderline forms, in which there is a higher prevalence of reactional neuritis and, consequently, greater disability (5, 6). This study corroborates previous neurophysiological assessments that also described a greater prevalence of sensory abnormalities, while motor impairment occur more frequently after treatment or in association with reactional episodes (5, 6, 19–22).

In this study, we confirmed a consistent distribution of serological and molecular results according to clinical forms, reinforcing the need to incorporate these tools into clinical practice, favoring early diagnosis. Furthermore, we also demonstrate that the impairment of the peripheral nerves can occur in all the clinical forms described above, but in a non-uniform way. In general, patients with the T form have strong cellular immunity against *M. leprae* antigens, but have low antibody production, while patients with the L clinical form do not have an effective cellular response against the bacillus. The borderline group presents intermediate responses, combining a high bacillary load with a cellular response that even triggers important neural damage (18).

The pathogenesis of peripheral nervous system involvement in leprosy is based on two main concepts around which neural damage is best understood: Schwann cell infection and the presence of perineural inflammation. Neural impairment may result from the direct effect of *M. leprae* infection, such as damage to neurofilaments, Schwann cell impairment and contact demyelination. Another mechanism suggests injuries resulting from the immune-mediated inflammatory process, including the action of antibodies, cytotoxicity, activation of cytotoxic T lymphocytes and others. Finally, neural impairment may result from edema and mechanical processes, that can lead nerve trunks more susceptible to compressive and even to ischemic damages (23–27).

The distribution of sensory deficits observed in leprosy neuropathy on neurological examination is defined as intradermal and truncal. The truncal pattern is defined by sensory and/or motor loss respecting the anatomical distribution of a specific nerve, while the intradermal sensory neuropathy was defined by the presence of sensory abnormalities in a region not respecting a specific nerve, branch or radicular territory. Only the truncal pattern is adequately evaluated in the electroneuromyographic study. Therefore, the number of affected nerves and the pattern described in the neurophysiological findings reinforce the truncal distribution of this neuropathy. The results described in this study corroborate with previous data in the description of the main trunks affected by the disease (5, 6, 19, 20, 28–30).

Additionally, *M. leprae* requires, in humans, an optimal temperature for replication averaged 77 to 91.4°F (25 to 33°C), and this is the estimated temperature of the superficial course of peripheral nerves (31, 32). This fact justifies the temperature-dependent pattern described in the disease. In our study, superficial nerves and those located at entrapment sites (ulnar at elbow and peroneal at head of the fibulae) were the most affected (5, 6, 31).

It is important to emphasize that even in the clinical forms in which the nerves were more diffusely affected, with multiple nerves affected by the bacillus, as in the BL and L forms, we did not find symmetry in the neurophysiological findings (5, 6). Despite the extent of the condition, a thorough electroneuromyographic evaluation can detect, in addition to asymmetries in the amplitudes of the SNAP and CMAP, which would justify a severe axonal impairment, the presence of asymmetries in distal latencies and conduction velocities, sometimes with temporal dispersion (21, 22, 29, 33–35). This data reinforces the presence of a confluent multiple mononeuropathy, however no length-dependent pattern of symmetrical polyneuropathy was found.

It is important to highlight that especially during the reactions, nerve conduction studies may reveal signs of demyelination, such as temporal dispersion, conduction block and pronounced reduction in conduction velocities, that are related to inflammatory activity, which may improve after steroid treatment (20).

The presence of intradermal involvement and the temperature-dependent pattern is essential in the clinical recognition of leprosy neuropathy, in all clinical forms (18, 29). In the multibacillary forms and with diffuse involvement (BV and V forms), the clinical investigation of these alterations corroborates the asymmetric pattern and not disease-dependent length. In addition, in a significant proportion of cases with the BT and BB forms, the electroneuromyographic evaluation is normal, and a detailed clinical evaluation is essential to investigate this pattern of neural involvement, which, combined with skin and laboratory alterations, favors the diagnosis.

The electroneuromyographic evaluation is very important in leprosy neuropathy, as it allows the recognition of neural dysfunction even before the onset of symptoms, since sensory and motor impairment was more evident in the ENMG than in the clinical evaluation (5, 19, 36, 37). Therefore, performing ENMG in patients diagnosed with leprosy is essential, as it allows not only the stratification of severity and pattern of peripheral neural impairment, but also the detection of initial forms, contributing to early diagnosis (38, 39).

Leprosy is a chronic, slowly progressive, disabling and challenging disease, not only in endemic countries, but also in those that have not reported new cases for many years. The world is experiencing a hidden endemic disease and recognition of the different forms of presentation is essential for early diagnosis and breaking the chain of disease transmission. This study reinforces the fact that leprosy is a spectral disease and that there is not a single laboratory test sufficient to guarantee the diagnosis. Furthermore, other tools that also fulfill the function of detecting neural damage, such as ultrasound of peripheral nerves, may also be useful. In fact, the control of the disease will only be possible through a broad clinical suspicion, laboratory and neurophysiological evaluation.

## Data availability statement

The original contributions presented in the study are included in the article/supplementary material, further inquiries can be directed to the corresponding author.

## Ethics statement

The studies involving humans were approved by Ethics Committee of the Federal University of Uberlandia (CAEE 45007721.7.0000.5152). The studies were conducted in accordance with the local legislation and institutional requirements. Written informed consent for participation was not required from the participants or the participants’ legal guardians/next of kin in accordance with the national legislation and institutional requirements.

## Author contributions

DS: Conceptualization, Data curation, Formal analysis, Investigation, Methodology, Project administration, Supervision, Validation, Visualization, Writing – original draft, Writing – review & editing. IB: Conceptualization, Data curation, Formal analysis, Investigation, Methodology, Validation, Visualization, Writing – original draft, Writing – review & editing. LG: Formal analysis, Methodology, Validation, Visualization, Writing – review & editing. DA: Formal analysis, Methodology, Software, Validation, Visualization, Writing – review & editing. AL: Formal analysis, Methodology, Validation, Visualization, Writing – review & editing. IG: Conceptualization, Data curation, Formal analysis, Funding–acquisition, Investigation, Methodology, Project administration, Resources, Supervision, Validation, Visualization, Writing – original draft, Writing – review & editing.
